# Higher chromosomal aberration frequency in products of conception from women older than 32 years old with diminished ovarian reserve undergoing IVF/ICSI

**DOI:** 10.18632/aging.202772

**Published:** 2021-03-26

**Authors:** Wanyu Zhang, Linghan Zhang, Yu Liu, Jing Li, Xiaolu Xu, Wenbin Niu, Jiawei Xu, Bo Sun, Yihong Guo

**Affiliations:** 1 Center for Reproductive Medicine, The First Affiliated Hospital of Zhengzhou University, Zhengzhou 450000, China; 2 Henan Province Key Laboratory of Reproduction and Genetics, Henan, China; 3 Department of Preimplantation Genetic Diagnosis, Center for Reproductive Medicine, The First Affiliated Hospital of Zhengzhou University, Zhengzhou, China

**Keywords:** diminished ovarian reserve (DOR), maternal age, single nucleotide polymorphism (SNP) microarray, first-trimester miscarriage, chromosome karyotype

## Abstract

Infertile women with diminished ovarian reserve (DOR) confront an increased miscarriage rate in assisted reproductive technology (ART). Genetic abnormality is the most important factor. However, the effects of DOR and female age on the molecular karyotype of products of conception (POCs) remain unknown. We analyzed POCs using a single nucleotide polymorphism (SNP) microarray from women with DOR who experienced first-trimester miscarriage in IVF/ICSI cycles. The SNP microarray revealed chromosomal abnormalities in 74.6% (47/63) of POCs, including trisomy in 83.0% (39/47). Chromosomal aberrations were more frequent in women older than 32 years old with DOR than in young women aged 20–32 years old (86.7% vs. 44.4%, P = 0.001). Univariate and multivariable analyses identified advanced age as a risk factor for chromosomal aberration-related miscarriage in women with DOR, with odds ratios of 8.125 (95% CI: 2.291–28.820, *P* = 0.001) and 5.867 (95% CI: 1.395–24.673, *P* = 0.016), respectively. The results showed that older women (older than 32 years old) with DOR had a high risk of miscarrying a chromosomally aberrant embryo/fetus, regardless of basal follicle-stimulating hormone (FSH), anti-Mullerian hormone (AMH), antral follicle count (AFC) and previous reproductive history. This finding indicates a novel cut-off value of age for women with DOR related to chromosomal aberration-related miscarriage.

## INTRODUCTION

Ovarian reserve (OR) refers to the development and ability of ovarian follicles to produce fertilized oocytes in the cortex. OR is used to describe female reproductive potential and predict the response to controlled ovarian hyperstimulation (COH) in the context of assisted reproductive technology (ART) [1]. Diminished OR (DOR) is generally defined by a reduced reproductive ability in older and younger women of reproductive age with regular menses compared to women of comparable age [2] due to age and to metabolic, genetic, autoimmune, enzymatic, iatrogenic, toxic and infectious causes [1]. DOR affects 6% to 64% of infertile women of different ages [3] and leads to a decreased pregnancy rate and increased miscarriage rate in women undergoing ART [4–6]. Patients with DOR have increased embryo aneuploidy [7–9]. Previous research has shown that first trimester pregnancy loss occurred in 15–20% of clinically recognized pregnancies, and 50% of pregnancy losses resulted from embryonic chromosomal aberrations [10]. This loss significantly increases the physical and psychological burden and economic losses of ART patients, especially young women with DOR, who expect a better outcome than their older peers.

The definition of DOR was not standardized according to the Practice Committee of the American Society for Reproductive Medicine (ASRM) [11]. Generally, OR is evaluated using basal follicle-stimulating hormone (FSH), anti-Mullerian hormone (AMH) and antral follicle count (AFC) in women with regular periods [12–14]. Studies related to the influence of the age of DOR patients on their offspring revealed conflicting results. Most analyses indicated that advanced age increased the risk of DOR [15] and chromosomal aneuploidy in embryos [16] simultaneously, and DOR was associated with low embryo morphology grades and increased aneuploid miscarriages and viable aneuploid pregnancies [15, 17, 18]. However, conflicting investigations have shown that female age was not a significant predictor of clinical miscarriage [19]; there was no relevance between DOR and oocyte quality [18, 20–23], and oocytes from women with DOR had similar potential for euploid blastocyst development. These studies focused on the effects of DOR and female age on chromosomal aberrations in oocytes or transferred embryos, respectively, while very few examined chromosomal abnormalities in products of conception (POCs). At the same time, most studies of this issue have failed to account for other risk factors for miscarriage that are influenced by age and DOR, such AMH, AFC and FSH. Therefore, little is known about the etiology of DOR in women experiencing first-trimester miscarriage.

To evaluate this question, we enrolled a retrospective database of patients with DOR who experienced first-trimester miscarriage and obtained POCs collected for single nucleotide polymorphism (SNP) microarray techniques to investigate the role of age and controversial markers relating to OR and ART characteristics in chromosomal aberration-related miscarriage in DOR patients. Notably, as the methods for evaluating the chromosomes of spontaneously aborted embryos develop, SNP microarray is a first-line method for the genetic detection of individual developmental disorders and congenital dysplasia by the International Standards for Cytogenomic Arrays Consortium [24]. It provides whole genome-wide screening with a higher resolution and detects unbalanced copy number variations (CNVs) with sizes greater than 100 kb.

## RESULTS

### Demographics of study subjects

There were 63 women with DOR in this study: 18 young (aged 20–32 years old) women and 45 old (aged over 32 years old) women. Table 1 presents the comparisons of different clinical characteristics and the ART strategies of enrolled couples in our study between the young and old groups. The larger proportion of female partners was in the old group (71.4%). There were no significant differences between the two maternal age groups in maternal-related parameters, such as BMI; serum basal E2, LH and thyroid-stimulating hormone (TSH) levels; gestational age at miscarriage (irrespective of fertilization method); fertilization method; the proportion of blastocysts transferred; and the number of embryos transferred. The median male age increased with the advancing age of their female partners, which was statistically significant in the two age groups (*P* < 0.001). The rates of prior gravidity (young group: 44.4%, old group: 73.3%, *P* = 0.040) and prior miscarriage (young group: 11.1%, old group: 44.4%, *P* = 0.039) were significantly different between the groups. In terms of reproductive potential, the young patients with DOR showed higher AMH (0.7 ± 0.2 vs. 0.5 ± 0.4, *P* = 0.040), higher AFC (3.8 ± 1.4 vs. 2.8 ± 2.1, *P* = 0.004), lower medians of FSH (9.3 vs. 10.5, *P* = 0.015) and more oocytes retrieved (7.2 ± 4.4 vs. 45.5 ± 2.8, *P* = 0.046) than the old group.

**Table 1:** Demographics of study objects grouped by age.

**Characteristics**	**Maternal age (years)**			***P*-value**
	**Total**	**20–32**	**≥33**	
No. of the cases	63	18 (28.6)	45 (71.4)	
**Paternal parameters**				
**Male age (years)**	33.6 ± 4.9	29.8 ± 4.0	36.0 ± 3.8	<0.001
**Maternal parameters**				
**BMI (kg/m^2^)**				NS
<25	49 (77.8)	14 (77.8)	35 (77.8)	
≥25	14 (22.2)	4 (22.2)	10 (22.2)	
**TSH (mIU/mL)**	2.1 ± 0.9	2.0 ± 0.7	2.1 ± 1.0	NS
**AMH (ng/mL)**	0.6 ± 0.4	0.7 ± 0.2	0.5 ± 0.4	0.040
**AFC**	3.1 ± 1.9	3.8 ± 1.4	2.8 ± 2.1	0.004
**Basal FSH level (mIU/mL)**	10.5 (8.7,12.4)	9.3 (8.3,11.5)	10.5 (8.9,12.9)	0.015
**Basal E2 level (mIU/mL)**	34.8 (25.4,41.9)	34.8 (28.5,41.9)	34.8 (24.4,52.4)	NS
**Basal LH level (mIU/mL)**	4.9 ± 2.2	4.6 ± 2.1	5.1 ± 2.3	NS
**Previous reproductive history**				
**Prior gravidity**				0.040
0	22 (34.9)	10 (55.6)	12 (26.7)	
1	21 (33.4)	6 (33.3)	15 (33.3)	
≥2	20 (31.7)	2 (11.1)	18 (40.0)	
**Prior miscarriage**				0.039
0	41 (65.1)	16 (88.9)	25 (55.6)	
1	18 (28.6)	2 (11.1)	16 (35.5)	
≥2	4 (6.3)	0	4 (8.9)	
**ART characteristics**				
**Fertility method (%)**				NS
IVF	47 (74.6)	11 (61.1)	36 (80%)	
ICSI	16 (25.4)	7 (38.9)	9 (20%)	
**No. of ovum pick-up**	6.1±3.5	7.2 ± 4.4	5.5 ± 2.8	0.046
**Day of embryos transferred (%)**				NS
D3	59 (93.7)	18 (100)	41 (91.1)	
D5	4 (6.3)	0	4 (8.9)	
**Gestational age at miscarriage (weeks)**	8.5 ± 0.6	8.4 ± 0.5	8.5 ± 0.7	NS
**SNP results**				
**Gender of POC (%)**				NS
male	35 (55.6)	10 (55.6)	25 (55.6)	
female	28 (44.4)	8 (44.4)	20 (44.4)	
**Chromosomal abnormality of POC (%)**				0.001
normal	16 (25.4)	10 (55.6)	6 (13.3)	
abnormal	47 (74.6)	8 (44.4)	39 (86.7)	

Abbreviations: Data are presented as numbers (%), means (95% CI), or median (25th, 75th percentile). BMI = body mass index; TSH = thyroid stimulating hormone; AMH = anti-Mullerian hormone; AFC = antral follicle count; FSH = follicle-stimulating hormone; LH = luteinizing hormone; ART = assisted reproductive technology; IU = international unit; IVF = *in vitro* fertilization; ICSI = intracytoplasmic sperm; SNP = single nucleotide polymorphism; POC = products of conception; NS = not significant; CI = Confidence interval.

### Results of the SNP array analysis of POCs

SNP microarray analyses of 63 chorionic villus samples enrolled in our study identified 47 cases (74.6%) with chromosomal aberrations, indicating that the abnormalities were predicted to be causative of the miscarriage. The following chromosomal abnormalities were detected: 83.0% trisomy (39/47); 8.5% triploid (4/47); 4.3% monosomy (2/47); 2.1% structural abnormalities (1/47); and 2.1% mosaicism (1/47) (Table 2). The rate of chromosomal abnormality of POCs was significantly different between the young and old women with DOR (44.4% vs. 86.7%, *P* = 0.013). The sex distribution of POCs in the young group was similar to that in the old group (male POCs: 55.6% vs. 44.4%, *P* = 1) (Table 1).

**Table 2:** Spectrum of abnormal chromosomal karyotype: type and variations between maternal age groups.

**Variables**	**Maternal age (years)**		**Total frequency (%)**
	**20–32 (*n* = 18)**	**≥33 (*n* = 45)**	
**Trisomy**			83.0 (39/47)
single	4	32	
double	0	3	
**Triploidy**	2	2	8.5 (4/4)
**Monosomy**	1	1	4.3 (2/47)
**Structural abnormality**	1	0	2.1 (1/4)
**Mosaicism**	0	1	2.1 (1/47)
**Total**	8	39	
**Total frequency (%)**	44.4 (8/18)	86.7 (39/45)	

**Figure 1:**
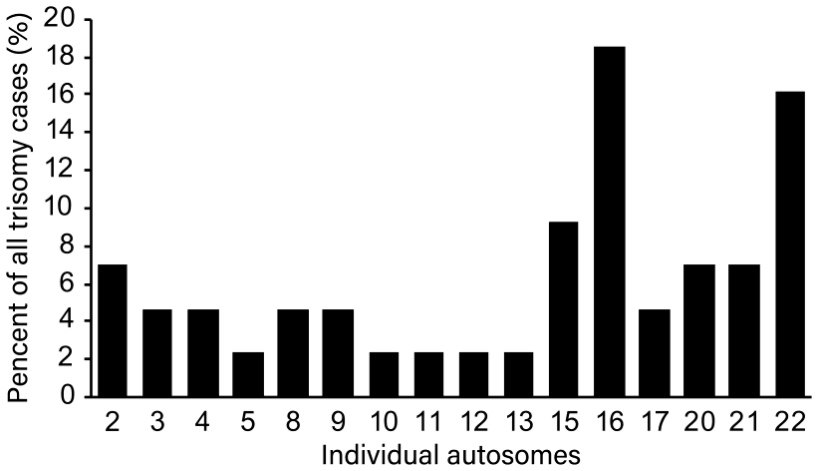
**Distribution of trisomy of individual chromosome among all trisomy cases.**

**Figure 2:**
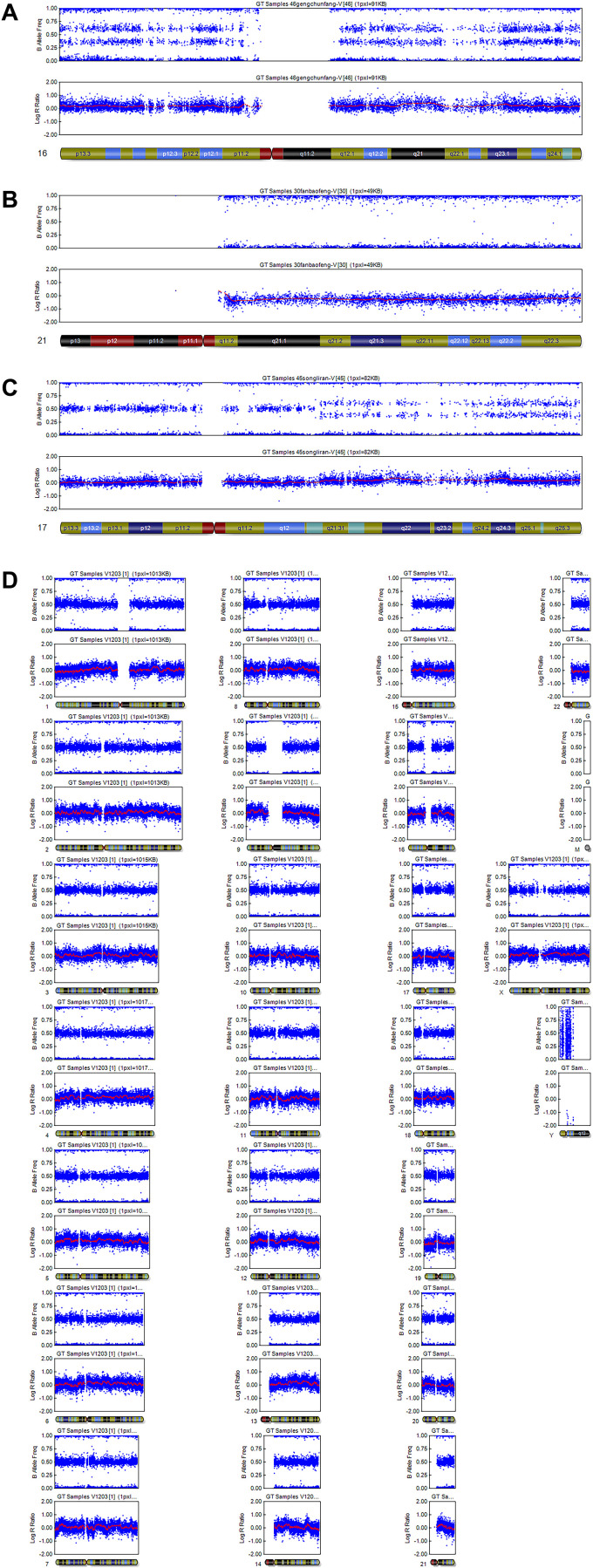
**Representative examples of SNP results.** (**A**) Single trisomy of chromosome 16. (**B**) Monosomy of chromosome 21. (**C**) Structural abnormality: duplication of chromosome 17. (**D**) Normal: arr (1–22) × 2, (X) × 2.

The most prevalent abnormality was trisomy. Single-chromosome trisomy was found in 36 cases and accounted for 92.3% (36/39) of all trisomies. Multiple trisomies (two or more chromosomes involved in trisomy) were observed in 3 chorionic villus samples, which constituted 7.7% (3/39). The most common trisomies were related to chromosomes 16 and 22 (18.5% and 16.2%, respectively), followed by 15 (9.3%), 20 (7.0%) and 21 (7.0%). No sex chromosome trisomy was observed, perhaps because of the small number of samples (Figure 1). Triploidy was found in 8.5% of samples (4/47). Three samples had a 69,XXY karyotype (75.0%), and 1 sample had a 69,XXX karyotype (25.0%). Monosomy was observed in 2 of 47 samples (4.3%), and both cases were on chromosome 21. There was 1 case of structural abnormality, related to duplication of chromosome 17, and 1 case of mosaicism, showing trisomy and monosomy. Representative examples of the SNP results of the POCs are shown in Figure 2.

### Age was a risk factor for chromosomal abnormality-related miscarriages among patients with DOR

The proportion of aberrant karyotypes among the young women (aged 20–32 years old) with DOR was significantly lower than that in the old (aged over 32 years old) women (44.4% vs. 86.7%, *P* = 0.001) (Table 1). Univariate and multivariate analyses were used to determine potential risk factors for chromosomal abnormalities of POCs. Univariate analyses indicated that the risk of chromosomal abnormality-related miscarriage was significantly higher in the old patients with DOR than in the young patients (odds ratio = 8.125; 95% confidence interval [CI], 2.291–28.820; *P* = 0.001). Compared to the control group of men aged 20–30 years old, male partners aged 31–40 years old (*P* = 0.019) and the female basal FSH level (*P* = 0.033) significantly correlated with chromosomally abnormal POCs. However, other variables, including female BMI, AMH with AFC, the number of oocytes retrieved and previous reproductive history, did not significantly correlate with chromosomally abnormal POCs (*P* > 0.05; Table 3).

**Table 3:** Logistic analysis of factors related to POCs with chromosomal aberrance.

**Variables**	**Univariable analysis**		**Multivariable analysis**	
	**Crude OR (95%CI)**	***P* value**	**Adjusted OR (95%CI)**	***P* value**
**Male age (years)**				
20–30	1^*^		1^*^	
31–40	4.100 (1.089, 15.441)	0.037	0.960 (0.099, 9.284)	0.930
**Gestational age at miscarriage (weeks)**	1.214 (0.649, 2.269)	0.544	0.593 (0.157, 2.237)	0.441
**Previous reproductive history**				
**Prior gravidity**	0.479 (0.224, 1.025)	0.058	0.497 (0.077, 3.218)	0.463
**Prior miscarriage**	0.349 (0.099, 1.228)	0.101	1.515 (0.135, 17.060)	0.737
**AFC**	1.221 (0.900, 1.656)	0.200	1.006 (0.622, 1.403)	1.627
**AMH**				
<1	1^*^		1^*^	
≥1	2.139 (0.420, 10.897)	0.360	2.352 (0.331, 16.728)	0.393
**Basal FSH level (mIU/L)**	0.796 (0.644, 0.982)	0.033	0.802 (0.602, 1.069)	0.132
**No. of ovum pick-up**	1.100 (0.908, 1.333)	0.331	0.931 (0.700, 3.218)	0.463
**Female age group**	8.125 (2.291, 28.820)	0.001	5.867 (1.395, 24.673)	0.016

^*^This variable functions as an indicator. Other categories of the same variable were compared with it. Abbreviations: AMH = anti-Mullerian hormone; AFC = antral follicle count; FSH = follicle-stimulating hormone; OR = odds ratio; CI = confidence intervals; IU = international unit.

Multivariate logistic regression, which was performed to adjust for potential confounding factors, showed that, for POCs with chromosomal abnormalities in women with DOR, female age was an independent risk factor, while male age and female basal FSH level were no longer statistically significant when adjusted for other clinical parameters. POCs from women with DOR older than 32 years old were more likely to develop abnormal karyotypes (adjusted odds ratio = 5.867; 95% confidence interval [CI], 1.395–24.673; *P* = 0.016).

**Figure 3:**
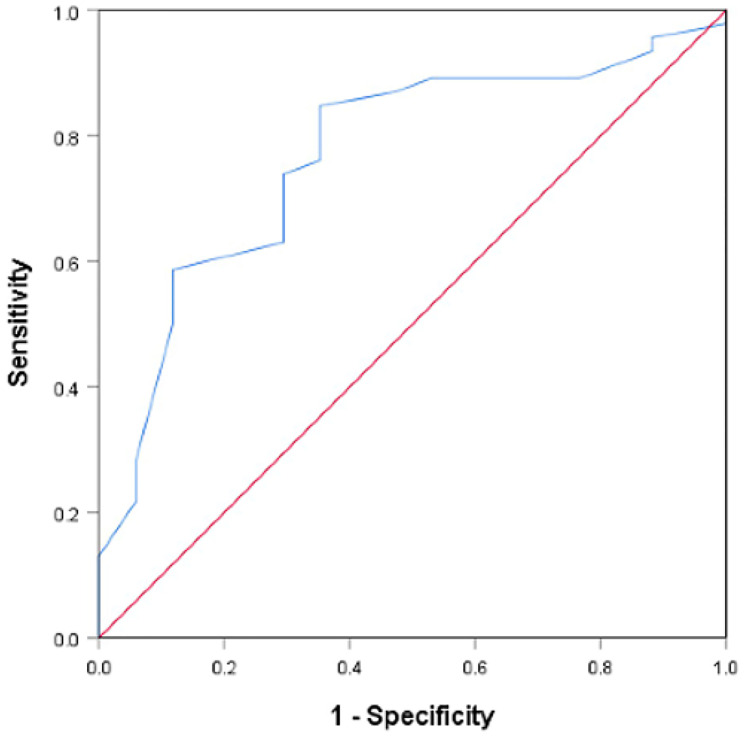
**Receiver operating characteristics curve of the predictive utility of female age for chromosomal abnormalities** among women with DOR (area under the curve (AUC) = 0.769, 95% CI: 0.639–0.898, *P* = .001).

The evaluation of age in the recognition of chromosomal aberration-related first-trimester miscarriage showed good performance and yielded an area under the curve (AUC) of 0.769 (95% CI: 0.639–0.898, *P* = 0.001; Figure 3). Notably, 32 years of age had sensitivity of 84.8% and specificity of 64.7%, while 30 and 35 years old presented sensitivity and specificity of 84.4% and 25.0% and 56.3% and 75.0%, respectively.

## DISCUSSION

### Lower chromosomal abnormality frequencies in POCs of young patients (aged 20–32 years old) with DOR

Our study demonstrated that age is an independent risk factor for chromosomal abnormality after adjustment. The aberration-related miscarriages among women with DOR were more frequent in the old group (older than 32 years old) than in younger women (20–32 years old). The exact mechanism by which advanced age (older than 32 years old) relates to higher chromosomal abnormalities in POCs remains elusive. The decline in fertility among women with DOR is related to age and DOR simultaneously. Age-related decline is definitely characterized by a quantitative and qualitative decline in OR and adverse pregnancy outcomes due to mitochondrial dysfunction, telomere shortening, impaired DNA repair, epigenetic changes and metabolic/energetic disorders [25]. Previous researchers have focused on the effects of maternal age on chromosomal abnormality in eggs or transferred embryos, while we mainly examined the effect of female age on abnormal chromosomes in miscarried fetuses. We believe this current study provides a new view of the strategy of diagnosis and treatment regarding the link between age of women with DOR and fetal development. Furthermore, age could influence the pathogenesis and degree of DOR. Previous studies have demonstrated the association between age and DOR from clinical data and molecular mechanisms. Young patients with DOR have a significantly greater likelihood of acquiring transplantable embryos and high-quality embryos than old patients with DOR, and the clinical pregnancy outcome was good once eggs were acquired [26]. Woo *et al*. [27] identified different expression profiles of miRNAs that regulate genes related to reduced oocyte quality only in young women with DOR, including miRNAs involved in the WNT, TGF-b, P13K-Akt, and MAPK signaling pathways in granulosa cells (GCs). Similarly, Skiadas *et al*. [28] confirmed altered gene expression in GCs only among young women with DOR. We identified female age as an important factor leading to the chromosomal abnormality of POCs and speculate that the mechanisms governing quality parameters and follicular depletion might be divergent in young and old women with DOR.

**Figure 4:**
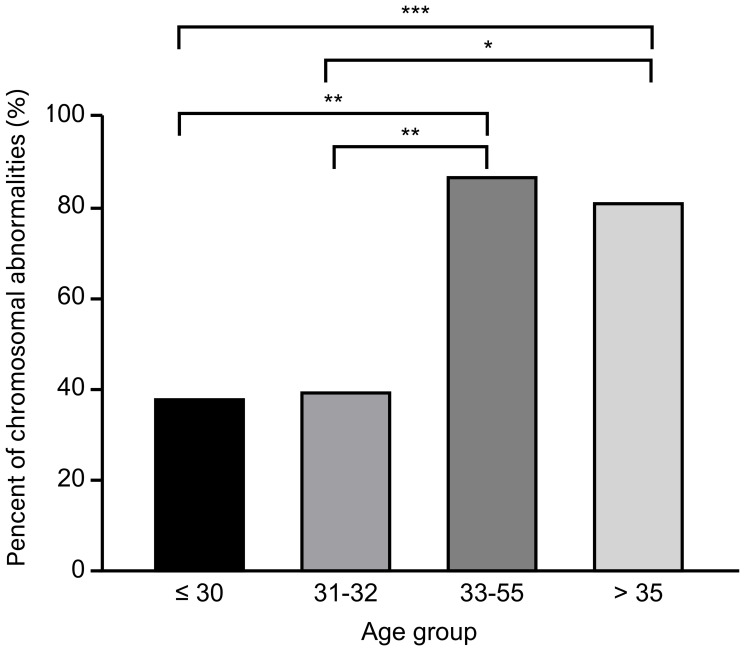
**Distribution of chromosomal abnormalities in products of conception in different age groups.**

In addition, we identified 32 years old as a cut-off value for the prediction of aneuploid POCs for several reasons. (i) Receiver operator characteristic (ROC) curve. The ROC curves demonstrated the predictive utility of female age (AUC = 0.769). Female age exhibited a cut-off value of 32 years old and good sensitivity and specificity of 84.8% and 64.7%, respectively. Compared with 32 years old, 30 and 35 years old, which are important ages in fertility, presented poor sensitivity and specificity of 84.4% and 25.0% and 56.3% and 75.0%, respectively. (ii) Previous research. A recent study [29] reported that the chromosome errors (aneuploidy) in human eggs followed a U-shaped curve after chromosome segregation in human oocytes from females aged 9 to 43 years old, in which the group aged 20–32 years old had the smallest proportion of aneuploidy MII oocytes, and maternal age was the only significant factor that affected the aneuploidy of oocytes, consistent with our results. (iii) Other investigation of data from our reproductive center. There is no currently accepted medical definition of advanced age relevant to aneuploid miscarriages in women with DOR. Researchers used to define 30 and 35 years old as cut-off values of age. We investigated 63 POCs using a SNP microarray from women included in our study further and divided them into <30, 30–31, 32–34, ≥35–year-old group. The results showed that there was no significant difference between each pair of groups (<30 years old vs. ≥30 years old; <35 years old vs. ≥35 years old) (Figure 4). In addition, the data in our center investigated by Li *et al*. [30] showed that there was no significant difference in the rate of karyotypic abnormalities between the <30-year-old group and the 30- to 35-year-old group among women with normal OR. Our study indicated that chromosomal abnormalities of POCs might be primarily due to advanced age and relate to a new and younger cut-off value of age (32 years old). We speculate that women with DOR are more sensitive to advanced age compared with women with normal OR, and the impact of age on chromosomal aberrations might be greater than that of DOR.

### Reproductive markers were not risk factors for a chromosomally aberrant fetus

In our study, several reproductive markers, such as AMH, AFC, basal FSH level, the number of oocytes retrieved and previous reproductive history, including prior gravidity and miscarriage, were significantly different between young and old patients with DOR in our study. The advanced age group showed lower AMH, AFC and number of oocytes retrieved and higher FSH, indicating poorer reproductive conditions. However, these factors were suggested not to be associated with abnormal chromosomes of chorionic villi after multivariate analysis. AMH, AFC, and basal FSH levels might reflect the quantity and quality of follicles and predict the number of transferred embryos through ovarian response to COS (controlled ovarian stimulation, COS). We included standardized and centralized measures of these reproductive markers, which were paired to a single IVF/ICSI-ET cycle leading to a clinical pregnancy to reduce the impact of subjectivity of the operator and within 1 year to weaken the influence of age-dependent variations. Furthermore, because the relationships of AMH, AFC and basal FSH levels with the number of retrieved oocytes have been proved, our study included the number of retrieved oocytes in multivariable analyses. Plante *et al*. [31] found that AMH levels did not differ between women with an aneuploid fetus and women with a euploid fetus. Notably, one study [17] confirmed a higher risk of abortion after IVF in women with low AMH (≤1.6 ng/ml) but only in older women (i.e., >34 years old). Another study found that AFC was not associated with any clinically significant increase in the risk of IVF pregnancy loss among women younger than 35 years of age [32]. Several studies were unable to confirm any independent association of AMH, AFC, basal FSH levels and the number of previous pregnancies and spontaneous miscarriages with aneuploidy in individuals who experienced pregnancy loss [20, 33–35], consistent with our study. Previous studies confirmed that DOR, as defined by baseline FSH, was not associated with any clinically significant increase in the risk of IVF pregnancy loss in women younger than 35 years old, suggesting that good quality oocytes could be obtained from young patients with DOR with high FSH levels. Our study showed no relationship of AMH, AFC, basal FSH level, the number of oocytes retrieved and previous reproductive history with chromosomal abnormalities in POCs, while the mechanism remains unclear, and prospective study of large samples is needed.

### Male age was a suspected risk factor

Male age was significantly different between the two groups due to female age, and it was a suspected risk factor for chromosome aneuploidy of POCs. Studies have often found conflicting results. Antonarakis [36] showed that only 5% of fathers contributed to trisomy 21 in their offspring, and male factors did not influence aneuploidy rates once a blastocyst was obtained [37]. However, a recent study found that male partners aged 20–24 years old showed a higher risk and those aged 30 years old or older had negligible effects on chromosomal aberration-related miscarriages compared to the effects of being 25–29 years old. Notably, our study excluded men older than 40 years old and/or with an abnormal karyotype to reduce the effect of male age. As a result, male age likely did not affect our results.

### Different age indicates different diagnoses and treatment focuses in women with DOR

Based on our study, we suggest diagnosing and treating first-trimester abortion differently in women with DOR at different ages. For young women (≤32 years old) with DOR, clinical doctors could primarily focus on maternal factors, such as immune, endocrine, infectious and prethrombotic states, and increase confidence once oocytes are obtained. However, for older women (older than 32 years old), there is a high risk of first-trimester abortion due to chromosomal aberrations of the embryo. Genetic consultation and preimplantation genetic testing of aneuploidies (PGT-A) might be advisable in future clinical practice.

### Strengths and limitations

To our knowledge, this report is the first study to show the effects of female age on chromosomal aberrations of POCs in women with DOR as defined by serum AMH and basal FSH levels simultaneously. Our study divided patients innovatively according to the qualities of human eggs and found a novel cut-off value of age. Furthermore, we examined the POCs via SNP microarray, which is different from PGT-A, which is used generally in current studies. The former is applied on the POCs and aims to adjust diagnostic and treatment strategies for the next ART cycle among women experiencing miscarriage. The latter is a preimplantation genetic test for aneuploidy applied on blastocysts and to improve the success rate in current ART cycles. We excluded women with a history of recurrent pregnancy loss (RPL) (defined as ≥2), which correlates with embryo aneuploidy [38], to reduce confounding risk factors. However, there are several limitations of our study. First, our conclusions are limited due to the retrospective design involving a single medical center, and the sample size was suboptimal. Second, consensus on the diagnostic criteria for DOR/OR is lacking. A previous study showed that the predictive value of OR screening tests could be low in a younger population [39]. Third, the database did not include occupational characteristics, environmental information, psychological conditions or lifestyle data, which could contribute to miscarriage [40].

## MATERIALS AND METHODS

### Study cohort and selection criteria

**Figure 5:**
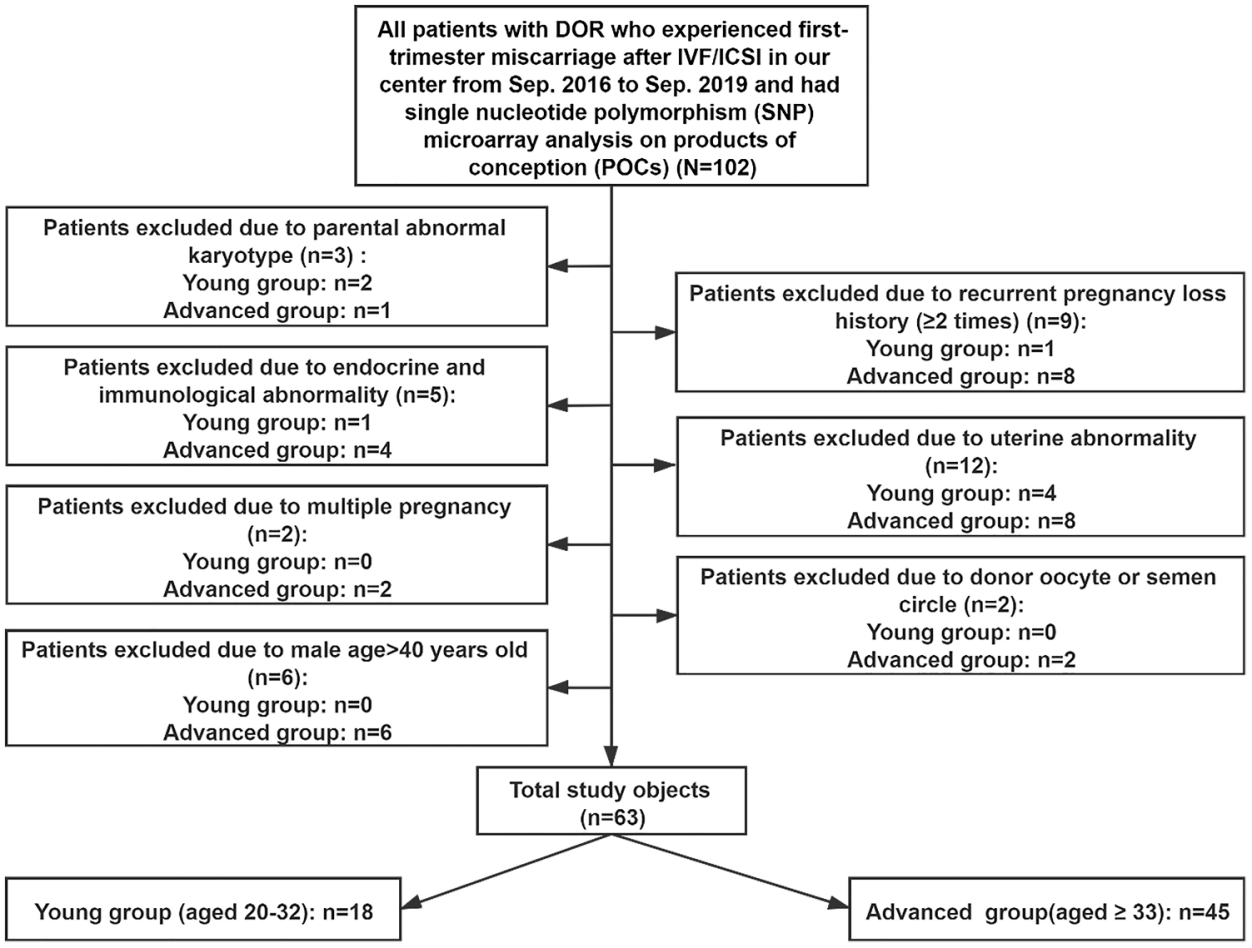
**Study inclusion and exclusion.**

We retrospectively enrolled 869 patients who experienced involuntary first-trimester miscarriage after ART and were treated with dilation and curettage (D&C). The aborted villous and embryonic tissues were transferred to a preimplantation genetic diagnosis center for genetic analyses from September 2016 to September 2019. The data were based on the Clinical Reproductive Medicine Management System/Electronic Medical Record Cohort Database (CCRM/EMRCD) in the Reproductive Medicine Center of the First Affiliated Hospital of Zhengzhou University and Henan Key Laboratory of Reproduction and Genetics. The committee in our hospital approved this study, and all of the patients provided written informed consent at their first consultation. A total of 104 patients defined as having DOR by the Federal Register Notice according to FSH >10 mIU/ml and/or AMH <1.0 ng/ml [41] were included. As shown in Figure 5, we excluded couples with abnormal chromosome karyotypes: females with a history of RPL (defined as ≥2) [10]; females with immunological abnormalities; couples with endocrine disorders in either partner, such as polycystic ovary syndrome (PCOS), thyroid dysfunction, diabetes, congenital adrenal hyperplasia, 21-hydroxylase deficiency and hyperprolactinemia; patients with uterine abnormalities, such as endometriosis/adenomyosis, submucous myoma or genital malformation; multiple pregnancies; cycles involving donor oocytes or sperm; and males older than 40 years old. Parental congenital and/or chronic diseases were taken into consideration, but none were detected. According to a recently published study of aneuploid human eggs [29], the resulting 63 participants were divided into a young group (aged 20–32 years old) and an old group (older than 32 years old). The numbers of participants were 18 and 45, respectively. For all of the patients, clinical information was obtained, including paternal characteristics (i.e., age, body mass index (BMI), and basal sex hormone levels), ART process (i.e., fertilization method and categories of embryos transferred) and results of genetic analyses.

### ART protocol

Among the patients included in our study, 47 underwent *in vitro* fertilization (IVF), and 16 received intracytoplasmic sperm injection (ICSI). A previous study showed that different ART protocols produced no significant differences in the molecular karyotype of POC tissues [30]. For IVF/ICSI, ovarian stimulation was performed with a standard long protocol using gonadotropin releasing hormone (GnRH) agonists to prevent a premature luteinizing hormone (LH) surge and gonadotropins to stimulate follicle growth. When the diameter of the maximal follicle was greater than 20 mm, and more than 2/3 of the total follicles were >16 mm, human chorionic gonadotropin (hCG) was provided according to the serum FSH, LH, E2 and P levels during the cycles. Oocyte retrieval was performed under ultrasonic guidance 36–38 h later. Serum β-hCG levels were monitored on days 14 and 18. The outcome of pregnancy was defined by the detection of a gestational sac with a fetal heartbeat in the uterine cavity via ultrasound. Early missed abortion was defined by the absence of fetal cardiac pulsation in the uterine cavity after confirmation of clinical pregnancy.

### DNA extraction and SNP microarray analysis

Chorionic villi from patients experiencing early missed abortion and undergoing D&C were sent to the preimplantation genetic diagnosis center for genetic analysis. Expert technicians cleaned the tissue with phosphate-buffered solution (PBS) to remove decidua and coagulated blood to avoid maternal genome contamination [42] and stored the samples at –80°C for DNA extraction.

Fresh DNA was extracted using a QIAamp DNA Mini Kit (Qiagen, Hilden, Germany), quantitated using Nanovue Plus (GE, Fairfield, CT, USA), and stored at -20° in preparation for subsequent SNP array analysis.

We used a Human CytoSNP-12v.21 Array (Illumina, San Diego, CA, USA) to detect molecular karyotypes and Genome-Studio (Illumina 2011) and Karyo-Studio software, version 1.4, to analyze the raw data. CNVs were mapped in the Database of Genomic Variants (DGV) (http://dgv.tcag.ca/dgv/app/faq) to identify candidate pathogenic CNVs. At least two independent technicians analyzed the data using strict criteria.

### Statistical analysis

Statistical analyses were performed using SPSS software, version 22.0 (IBM Corp., Armonk, NY, USA). For demographics of the study subjects, normally distributed continuous variables are presented as the means ± SDs, and differences between groups were assessed using Student’s *t*-test. Continuous variables with skewed distributions are represented as medians (P25, P75) and were compared using the Kruskal-Wallis test. Categorical variables are expressed as frequencies (percentages) and were compared using the chi-square test. Logistic regression analysis was performed to identify the risk factors for chromosomal aberration of POCs in early pregnancy loss when adjusting for several confounding factors. Two-sided *P* values less than 0.05 were considered significant.
